# Xenobiotic Metabolism: The Effect of Acute Kidney Injury on Non-Renal Drug Clearance and Hepatic Drug Metabolism

**DOI:** 10.3390/ijms15022538

**Published:** 2014-02-13

**Authors:** John Dixon, Katie Lane, Iain MacPhee, Barbara Philips

**Affiliations:** 1 General Intensive Care Unit, St. George’s Hospital, London SW17 0QT, UK; E-Mails: johndixon3@nhs.net (J.D.); klane@sgul.ac.uk (K.L.); 2 Division of Clinical Sciences, St. George’s, University of London, London SW17 0RE, UK; 3 Renal Medicine, St. George’s Hospital, London SW17 0QT, UK; E-Mail: imacphee@sgul.ac.uk

**Keywords:** acute kidney injury, cytochrome P450, drug metabolism, pharmacogenetics, pharmacokinetics, CYP3A

## Abstract

Acute kidney injury (AKI) is a common complication of critical illness, and evidence is emerging that suggests AKI disrupts the function of other organs. It is a recognized phenomenon that patients with chronic kidney disease (CKD) have reduced hepatic metabolism of drugs, via the cytochrome P450 (CYP) enzyme group, and drug dosing guidelines in AKI are often extrapolated from data obtained from patients with CKD. This approach, however, is flawed because several confounding factors exist in AKI. The data from animal studies investigating the effects of AKI on CYP activity are conflicting, although the results of the majority do suggest that AKI impairs hepatic CYP activity. More recently, human study data have also demonstrated decreased CYP activity associated with AKI, in particular the CYP3A subtypes. Furthermore, preliminary data suggest that patients expressing the functional allele variant CYP3A5*1 may be protected from the deleterious effects of AKI when compared with patients homozygous for the variant CYP3A5*3, which codes for a non-functional protein. In conclusion, there is a need to individualize drug prescribing, particularly for the more sick and vulnerable patients, but this needs to be explored in greater depth.

## 1. Introduction

The liver is the organ responsible for the majority of drug metabolism through the actions of the cytochrome P450 (CYP) enzyme group (Phase 1 reactions) and the enzymes of conjugation (Phase 2 reactions) [1]. Although these enzymes are predominantly active within the liver, many may also be found in various other organs including, kidney, gastrointestinal tract and lung [2]. Their function within these tissues may be of great importance to the function of that organ but with the exception of enzymes within the gastrointestinal wall, it is thought that overall drug metabolism is predominantly determined by the liver enzymes because of their abundance. Cytochrome P450 activity within the gut may have a marked effect on the bioavailability of orally administered medicines, less so on parenterally administered drugs.

Drug elimination however, occurs mainly via the kidneys [1]. This may be the parent drug itself or active or non-active metabolites and excretion predominantly occurs through filtration of hydrophilic compounds. However, there may also be significant secretion and re-absorption of compounds within the renal tubules, the importance of which varies between drugs and may change markedly in critical illness [3]. Other organs involved in the elimination of drugs include; the liver (through biliary excretion), the gastro-intestinal tract, lung and skin.

Drug dosing in AKI (acute kidney injury) is usually based upon empiric principles or is extrapolated from data obtained from patients with chronic kidney disease (CKD). This rationale, however, is flawed because AKI and CKD are different clinical entities with different etiologies and, for example, AKI occurring in the context of multi-organ dysfunction [4] has a different inflammatory milieu to CKD. Causes of AKI are outlined in Table 1. Furthermore, the time course for disease progression and adaptability differs which may result in varying pharmacokinetic and pharmacodynamic responses to the same drug in time: the rapidly changing nature of AKI means that therapeutic drug concentrations may not be achieved, or, alternatively, drug toxicity may ensue. Nevertheless, some mechanistic processes may be shared between CKD and AKI. AKI is inherently difficult to study, so, interesting results from investigations in patients with CKD may be useful in informing targets for research in AKI. The impact of CKD on non-renal clearance, particularly upon the function of CYP enzymes is now well recognized [5–7] and recommendations for drug dosing and interactions have been adjusted accordingly by the Food and Drug Administration (FDA) [8] The activity of the CYP enzymes may be similarly impaired in critically ill patients [3,9] and evidence suggests that acute kidney injury (AKI) may be implicated [10,11]. This review will focus on the evidence of the impact of AKI on non-renal drug clearance, with particular reference to CYP activity and expression.

## 2. The Kidney as a Metabolic Organ

Although the kidney is largely thought of as the organ responsible for elimination of waste products, toxins and drugs from the body, it is in fact more complex and has many other functions. These include; water and electrolyte homeostasis, maintenance of plasma osmolarity, acid-base balance, and the production and secretion of hormones, e.g., renin, erythropoietin, 1,25-dihydroxyvitamin D3 [12,13]. Some functions may be more important in pathological states [4], such as catabolism of peptide hormones and gluconeogenesis in fasting conditions. The kidneys receive 25% of the cardiac output under normal conditions and have high oxygen needs, largely caused by the energy required for the reabsorption of sodium ions in the proximal tubules [14].

As renal function declines, each of the kidney’s functions is affected, including clearance of drugs and their metabolites, although different functions may decline at different rates.

## 3. Importance of AKI (Acute Kidney Injury)

AKI is a clinical syndrome, defined in recent Kidney Disease Improving Global Outcomes (KDIGO) guidelines as “*an abrupt decrease in kidney function that includes, but is not limited to, acute renal failure*” [15]. AKI is defined according to three stages of severity: Stage 1 is defined by a rise in serum creatinine (SCr) of >1.5 times the baseline over the preceding seven days, a rise >26.4 μmol/L over the previous 2 days, or <0.5 mL/Kg/h urine output for >6 h; Stage 2 is defined by SCr > 2.0 the baseline, or urine output <0.5 mL/Kg/h for 12 h; and Stage 3 is defined by SCr greater than three times the baseline, initiation of renal replacement therapy, increase of SCr to >354 μmol/L or urine output <0.3 mL/Kg/h for >24 h or anuria for >12 h. An important feature of KDIGO criteria is that it defines AKI by relatively small increases in SCr. Retrospective observational cohort studies of patients with AKI reveal that small increases in SCr (>26.4 μmol/L) are associated with increased mortality when compared with patients without a change in renal function or patients with CKD [16]. The fact that relatively small changes in renal function affect mortality adds strength to the proposal that increased mortality is not merely due to retention of uraemic toxins, and that an underlying pathophysiological process may be affecting mortality in AKI.

There are several limitations to creatinine-based definitions of AKI; the most important are reduced sensitivity in patients with CKD [17] and reduced formation of SCr in critically ill patients [18].

Within critical care units, hypo-perfusion is the commonest cause of AKI [19], resulting in a mixture of pre renal failure and intrinsic AKI due to acute tubular necrosis (ATN). Once AKI develops, patients may experience some clinical effects generic to all causes of AKI, regardless of its aetiology, in addition to any effects specific to the illness causing AKI in that individual. It has not yet been established, however, whether different etiologies of AKI result in different clinical effects predominating.

AKI is a common complication of critical illness, and AKI requiring renal replacement therapy alone accounts for approximately 9% of all bed-days in general adult critical care units [20] and considerably more if all severities of AKI are included. Mortality from AKI requiring renal replacement therapy is between 43.3% and 74.5% [20,21] and has remained unchanged over the last 40 years, despite advances in renal replacement therapy. The reasons for the poor outcome from AKI are unclear. It is possible that the poor outcome is due to the decreased renal excretion of drugs and toxins. Decreased excretion may be directly attributable to a reduced glomerular filtration rate (GFR), reduced tubular secretion or impaired renal metabolism of drugs. There is, however, emerging evidence that AKI also affects the clearance of drugs and toxins by other organs (*i.e.*, non-renal clearance; *Cl*_NR_). These may contribute to high mortality associated with AKI. The evidence for this will be evaluated later and placed into clinical context.

## 4. Pharmacokinetics in AKI

There is a general paucity of pharmacokinetic (PK) studies concerning drugs in AKI and it is suggested that this may be due to a lack of incentive by pharmaceutical companies to fund studies in AKI because this is not yet a requirement of the FDA [10]. This complicates our understanding of such changes and in critically ill patients this is further complicated by multi-organ effects and cross talk between organs. Pro and anti-inflammatory changes, kidney, liver and endothelial dysfunction, drug interactions, therapeutic interventions, perfusion abnormalities, intestinal atrophy or gut dysmotility (impairing absorption of enterally administered drugs) are amongst the many confounding factors affecting the PK of any drugs administered in critically ill patients [9]. The volume of distribution of a drug may be affected by changes to cardiac output and peripheral perfusion [22]; and depends upon the degree of protein binding, tissue permeability and lipid solubility [23]. Other influential factors during AKI include changes to blood pH, the impact of AKI on pKa of the drug and the effects of fluid shifts between body compartments [24]. Each of these aspects may be influenced by renal dysfunction.

Currently, most drug-dosing regimens in patients with AKI are extrapolated from patients with CKD and of the evidence from studies of PK in AKI most are obtained from animal studies or are *ex vivo* investigations using cell cultures or microsomal systems. Interestingly it is suggested that *in vitro* studies using homogenates of renal cells may vastly underestimate the impact of AKI on drug metabolic pathways. Drug metabolizing enzymes are sited regionally within the kidney, for example; CYP enzymes are situated within the renal cortex, prostaglandin synthase within the medulla, *N*-acetyl transferase within both cortex and medulla [25]. Homogenizing a whole kidney means that enzyme activity may be grossly underestimated.

AKI has many etiologies, each with differing effects, e.g., Gentamicin accumulation within the renal cortex results in toxicity [26], causing acute tubular necrosis, whereas Cyclosporine toxicity causes altered renal haemodynamics and vasoconstriction [27]. It is possible that different etiologies of AKI have differing effects on hepatic enzyme activity.

## 5. Organ Crosstalk

Non-renal clearance (*Cl*_NR_) in AKI differs from that observed in patients without renal impairment. Even drugs normally associated with predominantly renal clearance (e.g., vancomycin) appear to be impacted upon by change in *Cl*_NR_ in AKI [10] although the mechanisms are unclear. A possible explanation for non-renal effects of AKI is “*organ crosstalk*”, that is, in the case of the kidneys, the effect of AKI on the function of other organs. Organ crosstalk has been defined as “*the effects of one malfunctioning organ upon the function of another*” [28], and is usually associated with injurious effects. An often studied example is that of acute lung injury following AKI. AKI results in increased pulmonary vasculature permeability to albumin, erythrocyte sludging in lung capillaries, interstitial edema and an inflammatory cell infiltrate in affected lung tissue [29]. Interestingly, these changes have been observed in rat models following kidney-ischaemia reperfusion injury [30] but not following bilateral nephrectomies [31], perhaps supporting the argument that this phenomenon is not purely due to uremia alone. Inflammation may contribute to remote organ dysfunction in AKI and may be important in the development of AKI. Animal models of ischemic AKI have demonstrated an abundance of cell adhesion molecules, increased cytokine-chemokine expression, leukocyte trafficking, dysregulation of apoptosis and increased oxidative stress in distant organs [31].

## 6. Potential Mechanisms of How AKI Affects Non-Renal Drug Clearance

The major contributor to *Cl*_NR_ is clearly hepatic clearance (*Cl*_HEP_) determined by the following equation [32]:

(1) $$Cl_{\text{HEP}}=[Q\times (Cl_{\text{INT}}\times f_{\text{UB}})]/[Q+(Cl_{\text{INT}}\times f_{\text{UB}})]$$

AKI may theoretically impact on each aspect of *Cl*_HEP_. Changes to liver blood flow affect uptake and elimination of drugs with a high extraction rate [33]. How important this is in renal-hepatic cross talk is uncertain as the full impact of AKI on liver blood flow and the liver micro-circulation is unknown. What is known is patients with CKD [34] and sepsis [35] have preserved global hepatic blood flow, perhaps suggesting it is not the most important factor.

Changes to protein binding and, hence the unbound fraction of a drug, have been observed in critically ill patients, thus altering the amount of drug available for metabolism within the liver [36]. Decreased serum albumin is initially a consequence of redistribution into interstitial fluids and later contributed to by decreased synthesis. Albumin binds mainly neutral and acidic drugs [37]. An important example is phenytoin which is highly bound to albumin, but its free fraction is increased in trauma patients as the serum albumin decreases [3]. The direct impact of AKI on serum albumin concentrations is uncertain but given that AKI is a pro-inflammatory state it is likely that it has some effect [37].

The usual rate-limiting step of intrinsic hepatic clearance is CYP activity [1]. CYP3A is responsible for metabolism of over 50% of drugs and there are two predominant isoforms CYP3A4 and CYP3A5. Individuals who possess at least one allele for the wild type CYP3A5*1 gene (*i.e.*, both homozygotes and heterozygotes) produce a functional protein. People homozygous for CYP3A5*3 produce a non-functioning CYP3A5 protein which is the predominant form in 80% of Caucasian populations. The wild type predominates in Sub-Saharan Africans [38], 60% are homozygous for CYP3A5*1. Other CYP enzymes involved in drug metabolism in humans include CYP1A, CYP2C19, CYP2C9 and CYP2D6, together they account for 80% of all drug metabolism [39]. CYP3A activity is impaired in CKD [40] and in AKI [11,41,42]. The impact of renal impairment on other CYP enzymes remains uncertain and may vary.

The underlying mechanisms for the inhibition of CYP enzymes are obscure. Parathyroid hormone, urea, and cytokines are all proposed as potential mediators [43]. Serum fractionation experiments in rodents suggest a 10–15 kDa substance may be responsible [44], but its identity is yet to be established.

Although CYP activity is responsible for the majority of drug metabolism, other enzyme systems have a role.

Flavin-containing-mono-oxygenase (FMOs) oxidizes xenobiotics containing Nitrogen, Sulfur, or Phosphorus [45]. They catalyze some of the same reactions as CYP enzymes [46], although they often result in different metabolites with potentially different pharmacological actions. The physiological functions of FMOs are currently poorly understood. Genetic polymorphism has been observed in three of the five human expressed FMO genes, FMO1, FMO2 and FMO3 [45]. The consequences of the genetic polymorphisms on drug metabolism remain poorly understood. The impact of AKI on FMO gene expression and FMO activity remains unclear.

There have been no studies directly investigating the effect of AKI on mono-amine oxidase (MAO), however, administration of pargyline, an irreversible MAO inhibitor, to a rat model of renal ischemia-reperfusion resulted in decreased tubular apoptosis and necrosis and increased proximal tubular cell proliferation [47]. This study demonstrated a central role of MAO in mediating the production of reactive oxygen species, which contribute to ischemia-reperfusion injury.

We have been unable to find in vitro, animal, or human studies investigating the impact of AKI on other enzyme systems, such as alcohol dehydrogenase, epoxide hydrolase, prostaglandin synthase or conjugation (Phase II) pathways.

Hepatic drug clearance also depends on drug transport systems including; organic anion transporter proteins (OATPs) which control uptake of drugs into hepatocytes [10] and P-glycoprotein which facilitates the elimination of drugs and metabolites from hepatocytes into the bile or blood. This is discussed in greater detail in Section 8. The impact of AKI on these systems remains unclear.

## 7. Evidence for Kidney-Liver Crosstalk

Experiments performed in animal models of AKI suggest a significant effect of AKI upon the inflammatory response and subsequent hepatic function. An altered balance of anti-inflammatory cytokines (e.g., IL-4, IL-10) and pro-inflammatory cytokines (e.g., TNF-α, IL-1, IL-6) and neutrophil infiltration has been observed in mice [36,48–52], rats [53,54] and dogs [55] following renal ischaemia-reperfusion injury and bilateral nephrectomy. IL-6 is a possible mediator of down-regulation of CYP activity [56]. In hepatic cell culture studies, several CYP isoforms have been down-regulated by IL-6 [57], but the clinical implications of this remain untested. AKI causes IL-6 to increase earlier and faster in sepsis [58], following cardiac surgery [59] and during acute lung injury [60]. IL-6 also activates the hypothalamo-pituitary axis, causing increased cortisol, which is an endogenous substrate of CYP enzymes and may lead to competitive inhibition [61]. This effect, however, is difficult to test because of the variability of cortisol in the critically ill, and because increased cortisol may induce CYP3A synthesis. Decreased activity of superoxide dismutase and catalase (both anti-oxidants) and increased malondialdehyde and transaminases concentrations have also been observed, implying that some of the observed changes in the liver may be due to oxidative stress [54].

The majority of animal studies investigating CYP activity demonstrate reduced hepatic drug metabolism, particularly when investigating CYP3A activity, however, some disparities exist. No change in Clarithromycin [62] or Telithromycin [63] metabolism via CYP3A was observed following Uranyl nitrate-induced AKI in rats, whereas decreased metabolism of Etoposide [64] and Losartan [65] metabolism via the CYP3A enzyme was demonstrated following Uranyl nitrate-induced AKI in rats. CYP3A activity was also reduced following Cisplatin-induced AKI in rats, as demonstrated by elevated Tacrolimus [66] and Quinine [67] concentrations. Gentamicin-induced AKI did not change CYP3A-mediated Cyclosporine metabolism in rats [68], whereas it was reduced following renal-ischaemia-reperfusion injury in rabbits [69]. AKI induced by bilateral ureteric ligation or by Uranyl nitrate did not result in altered CYP-2D6 mediated metabolism of Metoprolol in rats [70,71]; no change in CYP2D6-mediated metabolism of Propranolol was observed in rats when AKI was induced by Cisplatin [66], but elevated Propranolol concentrations were observed, implying reduced CYP2D6 activity, when AKI was induced by bilateral ureteric ligation in rats [72]. Increased metabolism of Theophylline, via the CYP2E1 enzyme, was observed following Uranyl nitrate-induced AKI in rats [73], and increased CYPD2C-mediated metabolism of Tolbutamide was observed following glycerol-induced AKI in rats [74].

AKI may induce modifications in the transcription and translation of CYP enzymes; however, changes in mRNA-CYP protein expression do not always result in altered enzyme activity. Decreased mRNA-directed expression of CYP2E1 was observed in AKI following renal-ischaemia-reperfusion-injury in rats [75], whereas it was increased following Uranyl nitrate-induced AKI [76].

It is difficult to directly apply data obtained from experiments in animals to humans. The apparent conflicting evidence may be due in part to the characteristics of the drugs studied and the model of AKI used. In addition, CYP isoforms are different between species and control of CYP expression may also differ. The changes in CYP activity that occur in one organ do not always occur in others (e.g., increased activity in intestine despite decreased activity of CYP3A4 in liver). While changes in hepatic drug metabolism was not always observed, it is possible that other pharmacokinetic changes may have occurred, for example, altered intestinal absorption or gut CYP3A activity, or altered protein-binding. Extrapolation of data from animal studies to humans requires extreme caution.

## 8. Transporters

Few studies have been performed in AKI, and all are in animal models or cell cultures. Interspecies differences exist, in their tissue distribution and subtypes. These need to be taken into account when extrapolating data to humans.

Renal drug clearance involves transport across the basolateral and apical tubular membranes. Transporter activity is a major influence on drug clearance. The main mechanisms involve (1) passive diffusion through the lipid cell layer, or via aqueous channels; (2) Carrier mediated transport via either active (ATP-dependent) transport or facilitated transport.

Drugs passively diffuse across cell membranes along a concentration gradient without expenditure of energy. Important factors affecting lipid diffusion are: the concentration, the surface area of the drug (greater diffusibility occurs with drugs that have a larger surface area), and the lipid-solubility of the drug. The lipid-solubility depends on the lipid-aqueous partition co-efficient (*i.e.*, how readily a drug can pass through a lipid membrane), the degree of ionization of the drug, which depends on the pKa of the drug and the pH of the surrounding cell medium. Aqueous diffusion occurs via aqueous pores along a concentration gradient. Drugs passing through aqueous pores are small (molecular mass < 30 kD) and water-soluble in solution. We have been unable to find data regarding the specific effects of AKI upon passive diffusion of drugs; however, it is possible that acidosis associated with AKI may impact on the lipid-aqueous partition co-efficient by altering pH of body fluids.

Carrier-mediated transport is important for drugs that are too large or too insoluble in lipid to diffuse through lipid membranes. Carriers are trans-membrane proteins and mainly located in renal tubules, the biliary tract, the blood-brain barrier and the gastrointestinal tract. Active transport occurs against a concentration gradient, is energy dependent with energy obtained from hydrolysis of ATP. Carriers are selective, may become saturated once a threshold has been reached, and may undergo competitive inhibition by another drug binding to the same receptor. One large family is the ABC (ATP binding cassette) and includes P-glycoprotein, or multidrug resistance type 1 (MDR1) transporter. P-gp is an ATP-dependent efflux pump expressed in the liver, kidneys and intestines [10]. It assists transportation of lipophilic compounds from inside cells to the bile, urine and intestinal lumen, assisting with clearance of the drug from the body. Increased P-gp expression was observed in the kidney of rats with AKI [77], but not their liver [78] or intestines [79]. Clearance of P-gp substrates was decreased, however, throughout the body, including via the liver, kidneys and intestines, implying global suppression of P-gp function during AKI. The effect of AKI on P-gp suppression may impact on clearance of drugs such as Digoxin, Methotrexate and Vincristine [80].

The organic anion transporters (OATs) and organic cation transporters (OCTs) are important in transferring drugs across cell membranes. OATs mainly occur in renal tubular basolateral membranes and enhance uptake of small organic anions from the peri-tubular plasma into renal tubular cells, by efflux across the apical membrane into the tubular lumen [10]. Decreased OAT-1 and OAT-3 mRNA protein expression was observed in rats with AKI [81]. The role of OATs in non-renal clearance has not yet been clarified; however, decreased activity during AKI may impact on clearance of drugs such as Methotrexate and NSAIDs [82]. We could find no literature regarding the effect of AKI upon organic cation transporter (OCT) activity.

## 9. Human Studies

To date, three published studies have investigated the effect of AKI on hepatic drug metabolism in humans [11,41,42]. Heinemeyer investigated the effect of AKI upon hepatic clearance of Cetriaxone in post-operative patients with pneumonia and AKI [42]. Delayed biliary excretion was demonstrated, however, the free-drug fraction differed between patients with AKI and those without. The underlying mechanisms were not explored, so the authors were unable to exclude sepsis as the cause of liver dysfunction, rather than AKI. A second study by the same group investigated the clearance of monomethylaminoantipyrine (MMAAP) in critically ill adults with AKI [41]. Significantly reduced Clearance of MMAAP was observed in patients with AKI compared to those without AKI.

Furthermore, a significantly reduced rate of appearance of its metabolites *N*-formylaminoantipyrine and *N*-acetylaminoipyrine was observed. It appears likely that the reduced hepatic metabolism was responsible for decreased rate of MMAAP clearance occurring in AKI, although the authors were unable to exclude other potential confounders, such as hypoxia, reduced cardiac output, reduced protein synthesis, or competitive antagonism by other drugs. However, in contrast to their previous study, less than 10% of subjects had septic shock, making sepsis unlikely as the sole cause of this phenomenon. More recently, our group has used an intravenous Midazolam probe to investigate CYP3A4 and CYP3A5 activity in critically ill patients with AKI [11]. A significant decrease in Midazolam elimination was observed in patients with AKI, and this effect appeared to be increased with prolonged durations of AKI. These findings were significant, despite the heterogenous population studied, perhaps implying a potent effect. Other potential confounders, such as acid-base balance and serum albumin concentration, were not significantly different between patients with AKI and those without. In addition, preliminary data suggests CYP function may be preserved in patients with AKI who expressed either homozygous or heterozygous functional allele variant CYP3A5*1 when compared with those who were homozygous for the splice variant CYP3A*5, which codes for a non-functional truncated protein. This could have important pharmacogenetic implications for patients with AKI but remains to be fully tested.

It is plausible that uraemic toxins may be responsible for the changes in CYP activity occurring during AKI, and it is also conceivable that removing potential toxins with renal replacement therapy or plasma exchange may reverse the non-renal clearance effects observed in AKI. To date, few studies have investigated this. In one study, patients with AKI had increased Telithromycin concentration and exposure (as measured by area under the curve), however, AUC approached that of healthy individuals within two hours of renal replacement therapy [83]. In another study, the 14C-Erythromycin breath test was used as a marker of CYP3A4 activity and 27% increase in activity was observed 2 h after initiation of renal replacement therapy [84].

## 10. Role of Intestinal Metabolism

The majority of drug metabolism occurs in the liver, although metabolism within the gut wall is important for some orally administered drugs. CYP3A accounts for 80% of small intestine drug metabolism but only 1% of total body CYP3A activity [85]. Some drugs depend on gut wall enzymes to convert a pro-drug to its active form. Uptake from the gut lumen occurs either by diffusion or active OAT-mediated transport, before being then passed into the portal circulation en route back to the liver or extracted back into the gut lumen. It is not know to what extent AKI impacts on this process, however, critical illness in general may impair absorption by altering gut perfusion, it may impair gut motility and may alter gut flora. We were unable to find any data on the effects of AKI on intestinal CYP2J2, the other abundant intestinal CYP enzyme.

## 11. Conclusions

Current drug-dosing guidelines and regimens in patients with AKI have several limitations. The majority use empiric principles or use data extrapolated from patients with CKD. The pitfalls of this are becoming more obvious and we have got to the stage where greater understanding of the wider implications of AKI is required in order to optimize drug treatment of who are very often very vulnerable patients. It is likely that we should be making adjustments for alterations in hepatic drug clearance during AKI. The mechanisms for any effect on non-renal drug clearance remain unclear but involve the accumulation of toxins (e.g., IL-6).

Our understanding is limited by small studies and extrapolation of data from animal studies and *in vitro* investigations. Metabolizing enzymes and transporter isoforms differ between species and interpretation of data from immortal cells lines, particularly concerning the expression of certain proteins is fraught with problems. Nevertheless evidence is accumulating that, as with CKD, AKI does have a significant impact on the hepatic metabolism of drugs and could also affect drug clearance in other organs (e.g., intestines). Clinical studies investigating the effect of AKI may be confounded by the existence of multiple pathological processes in critically ill patients with AKI that may also impair CYP and transporter activity (e.g., sepsis, trauma, burns). It is also likely that, once the pharmacokinetic effects of AKI have been accounted for, inflammatory mediators occurring during critical illness may influence the pharmacodynamic response to drugs.

In order to bring this field forward and improve drug prescribing on individual level, future studies need to elucidate mechanisms at the enzyme and mRNA levels and their clinical effects in patients with and without AKI. Furthermore, distinctions need to be made between gut, liver and kidney metabolism of the various CYP enzymes and transporters. The influence of other co-existent diseases that contribute to AKI needs to be accounted for and excluded if possible. Identification of putative uraemic toxins, and whether their removal by renal replacement therapy improves non-renal clearance, also needs clarification.

## Figures and Tables

**Table 1.** Causes of acute kidney injury (AKI).

Category of AKI	Mechanism	Causes
Pre-renal failure *Renal hypoperfusion*	Hypovolaemia/Hypotension	Haemorrhage, dehydration (diarrhoea and vomiting, heat), Osmotic diuresis (hyperglycaemia, iatrogenic), excessive diuretic use
	Redistributive shock	Sepsis, anaphylaxis, reduced plasma oncotic pressure in nephrotic syndrome, pancreatitis
	Poor cardiac function	Cardiogenic shock, severe sepsis,
	Renal vascular changes	Afferent arteriolar vasoconstriction (NSAIDs, ACE inhibitors, vasoconstrictors)

Intrinsic-renal failure *Damage to the renal parenchyma*	Glomerular damage	Primary or secondary glomerulonephritis (infective, autoimmune, inflammatory)
	Tubular damage	Ischaemia or nephrotoxins, sepsis
	Damage to the renal blood vessels	Haemolytic uraemic syndrome
	Interstitial damage	Nephrotoxins or infection, sepsis

Post-renal failure *Damage to the renal outflow of urine*	Obstruction within the upper renal tract	Stones or malignancy
	External obstruction of the upper renal tract	External compression due to a mass, constriction due to retroperitoneal fibrosis, Intra-abdominal compartment syndrome
	Obstruction to the lower renal tract	Bladder neck dysfunction, prostatic enlargement, uterine disease, obstructed catheters
